# Tracking Focal Adhesion Turnover: A Novel Reporter for FA-Phagy Flux

**DOI:** 10.3390/cells15030306

**Published:** 2026-02-06

**Authors:** Kuizhi Qu, Mengjun Dai, Ying Jiang, Sophie Liu, John P. Hagan, Louise D. McCullough, Zhen Xu, Yan-Ning Rui

**Affiliations:** 1 Department of Neurosurgery, McGovern Medical School, The University of Texas Health Science Center at Houston, Houston, TX 77030, USA; kuizhi.qu@uth.tmc.edu (K.Q.); mengjun.dai@uth.tmc.edu (M.D.); ying.jiang@uth.tmc.edu (Y.J.); liuckg@bc.edu (S.L.); john.p.hagan@uth.tmc.edu (J.P.H.); 2 Department of Neurology, McGovern Medical School, The University of Texas Health Science Center at Houston, Houston, TX 77030, USA

**Keywords:** focal adhesions, autophagy, FA-phagy, lysosome, new assay

## Abstract

Focal adhesions (FAs) are critical multi-protein complexes regulating cell adhesion, migration, and survival, and their dysregulation contributes to cancer metastasis and vascular diseases. Despite extensive research on FA formation, little is known about FA turnover, particularly its regulation by autophagy. This study introduces a novel tandem fluorescence reporter capable of tracking the entire FA-phagy flux, from autophagosome formation to lysosomal degradation. The reporter, based on a red–green fluorescence system with a lysosome-specific cleavage site, integrates seamlessly into endogenous focal adhesion complexes, demonstrating sensitivity and specificity to autophagy stimuli. Validated in multiple cell lines, the tool revealed dynamic FA-phagy responses to starvation-induced autophagy and the involvement of autophagy regulators such as *mTOR* and *ATG* genes. This versatile reporter provides a powerful tool for investigating FA-phagy mechanisms, with significant implications for cancer biology and vascular research.

## 1. Introduction

Focal adhesions (FAs) are dynamic, multi-protein complexes that serve as key structural and signaling hubs at the interface between the cell and its extracellular matrix [1]. They orchestrate cellular responses to mechanical and biochemical cues, influencing processes such as cell adhesion, differentiation, and survival [2]. Dysregulated FAs can cause diseases by promoting cancer metastasis through increased cell movement and invasion [3], and by disrupting the integrity of blood vessels, leading to vascular diseases such as aneurysms [4].

Despite extensive studies on the formation and assembly of FAs, relatively little is known about their turnover. FA turnover is crucial for processes such as cell migration and tissue remodeling [5]. Recent studies have highlighted the role of autophagy in the degradation of FAs [6,7]. Autophagy, a conserved catabolic process, encompasses three distinct pathways: macroautophagy, microautophagy, and chaperone-mediated autophagy [8]. Macroautophagy involves the sequestration of cytoplasmic cargo within double-membraned autophagosomes, which subsequently fuse with lysosomes for degradation [9]. Microautophagy involves the direct engulfment of cargo by lysosomes, and chaperone-mediated autophagy relies on specific recognition by a protein chaperone and translocation of cargo into lysosomes [10]. In general, autophagy maintains cellular homeostasis by recycling cellular components, responding to stress, and removing damaged organelles or protein aggregates.

Several publications have reported the contribution of selective macroautophagy to FA turnover. For instance, NBR1 and Cbl1 can serve as cargo receptors that bridge autophagosomes to focal adhesion complexes, thereby facilitating their lysosomal degradation [11,12]. In addition, SRC-mediated phosphorylation of paxillin enhances its recognition by autophagosomes [11]. Our group has advanced this field by coining the term “FA-phagy” in analogy to other forms of selective autophagy, such as “ER-phagy” [13]. FA-phagy refers to the selective autophagy-mediated degradation of focal adhesion complex. This process involves the recruitment of autophagosomal machinery to focal adhesions and subsequent autophagosome–lysosome fusion, enabling their lysosomal turnover in a selective manner.

Although some mechanisms were revealed in FA-phagy, it remains largely unknown due to the lack of a specific and sensitive assay for monitoring FA-phagy in cells. To our knowledge, most studies on FA-phagy have used LC3-related assays, such as GFP-LC3 colocalization with FAs [14]. However, FA-phagy is a multi-step process that includes autophagosome biogenesis, cargo recognition, autophagosome–lysosome fusion, and cargo degradation. Some FAs in GFP-LC3-labeled autophagosomes may not progress to the final step. Additionally, GFP fluorescence is quenched in acidic lysosomes [15], which may cause FAs already in autolysosomes to be overlooked due to the loss of GFP-LC3 signals.

Tandem fluorescent reporters have been widely used to monitor the entire autophagic flux in different selective autophagic processes. For example, an mCherry-GFP tag fused to RAMP4 has been applied to visualize ER-phagy flux under starvation conditions [16]. Similarly, tandem reporters have been coupled to other organelle-specific markers to detect ribophagy, pexophagy, and mitophagy [17,18,19]. Inspired by these prior studies, we fused the mCherry-GFP reporter to the focal adhesion protein zyxin. This reporter specifically tracks zyxin, a marker of mature focal adhesions that forms complexes with other focal adhesion proteins, such as paxillin and vinculin. To enhance the efficiency and specificity of mCherry-GFP separation during lysosomal degradation, we incorporated an LSCS linker sequence, which is preferentially cleaved by the lysosomal protease legumain [20,21]. This innovative tool allows dynamic observation of the entire FA-phagy process, providing new insights into the temporal and spatial dynamics of autophagy-mediated FA turnover. This approach may enable the identification of new modulators in the FA-phagy pathway and offer new insights into its roles in health and disease.

## 2. Materials and Methods

### 2.1. Plasmids

The pLVX-TetOne-Puro vector was purchased from Takara Inc. (Houston, TX, USA). and subsequently engineered to contain a customized polylinker harboring multiple 8-cutter restriction sites, including PacI and NotI, to enhance cloning flexibility. This modified vector was designated pLTO-PANBR. The full-length mCherry-LSCS-GFP-zyxin construct was inserted into pLTO-PANBR via PacI and NotI sites using standard PCR-based cloning methods. All constructs were confirmed by Sanger sequencing as previously described [22].

### 2.2. siRNAs

All stealth small interfering RNAs (siRNAs) were purchased from ThermoFisher Scientific (Waltham, MA, USA) and included siRNAs targeting atg5 (HSS114103 and HSS114104), and atg7 (HSS116182). Beclin 1 small interfering RNAs (siRNAs) were purchased from Sigma (WD01767152, Rockville, MD, USA). The effectiveness of each siRNA in knockdown experiments was validated through Western blotting.

### 2.3. Antibodies

S6K (9202), p-S6K-T389 (9205), 4E-BP1 (9452), p-4E-BP1-T37/46 (9459), Beclin 1 (3495), ATG5 (2630), and ATG7 (8558) were from Cell Signaling Technology (Danvers, MA, USA); Vinculin was from Santa Cruz Biotechnology (Dallas, TX, USA) (sc-73614); Paxillin was from BD Biosciences (San Jose, CA, USA) (610051); Zyxin was from Millipore (Billerica, MA, USA) (MAB2610); LC3 was from MBL (Woods Hole, MA, USA) (PM036); mCherry was from Novus biological (Centennial, CO, USA) (NBP2-25157); GFP was from Aves (Sugar Land, TX, USA) (GFP-1010); and GAPDH was from Santa Cruz Biotechnology (Dallas, TX, USA) (sc-32233).

### 2.4. Cell Culture

HeLa cells, COS7 cells, SHEP cells, NIH3T3 cells, and U2OS cells were cultured in Dulbecco’s Modified Eagle Medium (MT10013CV, Corning, Corning, NY, USA) with 10% fetal bovine serum. Inducible gene expression in HeLa was achieved through lentiviral infection. Lentiviruses were generated in HEK293T cells with the assistance of two other plasmids, pMD2.G (#12259, Addgene, Watertown, MA, USA) and psPAX2 (#12260, Addgene). For siRNA-mediated knockdown experiments, siRNAs were introduced into HeLa using a standard lipofectamine transfection protocol (L3000015, Invitrogen, Carlsbad, CA, USA). FA-phagy was induced by treating HeLa with Earle’s Balanced Salt Solution (EBSS) for 16 h.

### 2.5. Western Blot

Cells were treated with siRNAs or compounds in 60 mm dishes and lysed using Triton X-100 lysis buffer, as previously described [22]. The lysates were briefly sonicated and then centrifuged at 13,000 rpm for 30 min at 4 °C. Equal amounts of the resulting supernatants were mixed with 2× SDS sample buffer and resolved by SDS-PAGE. Proteins were subsequently transferred onto nitrocellulose membranes using a Bio-Rad (Hercules, CA, USA) mini transfer system. Membranes were blocked in 5% non-fat milk before incubation with primary antibodies (1:1000) and secondary antibodies (1:10,000). Fluorescent signals were visualized using the Odyssey Imaging System (LI-COR Biosciences, Lincoln, NE, USA).

### 2.6. Immunostaining

Cells were replated onto 8-well chamber slides (Fisher, Houston, TX, USA) at appropriate densities and subjected to the indicated treatments. Following fixation with 4% paraformaldehyde (Fisher, Houston, TX, USA) and permeabilization with Triton X-100 (Fisher, Houston, TX, USA), cells were blocked with 10% normal goat serum (Fisher, Houston, TX, USA) for 1 h at room temperature. Samples were then incubated with primary antibodies overnight at 4 °C, followed by staining with Alexa Fluor 488-, 594-, or 647-conjugated secondary antibodies (Invitrogen, Carlsbad, CA, USA). Coverslips were mounted using Prolong Gold antifade reagent (Invitrogen, Carlsbad, CA, USA), and images were acquired with a Nikon intravital confocal microscope (Melville, NY, USA).

### 2.7. Live Imaging of FA-Phagy by Confocal Microscopy

Cells were seeded at an appropriate density in a 35 mm dish (NC9268399, Fisher, Houston, TX, USA) and cultured in Dulbecco’s Modified Eagle’s Medium (MT10013CV, Corning, NY, USA). To induce reporter expression, 0.5 µg/mL of doxycycline was added, and cells were incubated at 37 °C with 5% CO_2_ for 24 h. Starvation was induced using Earle’s Balanced Salt Solution (SH3002902, Cytiva, Marlborough, MA, USA) for 16 h, with or without 100 nM Bafilomycin A1 (AAJ61835MCR, Fisher, Houston, TX, USA). Fluorescence imaging was performed using a Nikon A1R confocal microscope (Melville, NY, USA). LC3-positive puncta were quantified using Fiji/ImageJ (1.53-win-java8). For each condition, at least 116 cells from three independent experiments were analyzed. After background subtraction, LC3 puncta were identified by automated thresholding with size filtering to exclude noise. The number of LC3 puncta within each cell profile was counted, and the average number of puncta per cellular profile was obtained by dividing the total puncta count by the total number of cells analyzed under each condition. FA-phagy activity was assessed using a tandem mCherry-GFP–tagged focal adhesion reporter. Yellow puncta (mCherry+GFP+) represent focal adhesions prior to lysosomal degradation, whereas red-only puncta (mCherry+GFP−) indicate focal adhesions delivered to lysosomes where GFP fluorescence is quenched. Both GFP and mCherry signals were imaged in live cells and recorded using a Nikon confocal microscope (Melville, NY, USA). FA-phagy activation was quantified as percentage of the cells having red puncta under each condition.

### 2.8. Wound Healing

Cells were seeded at an appropriate density in a 35 mm dish (NC9268399, Fisher, Houston, TX, USA) and cultured in Dulbecco’s Modified Eagle’s Medium (MT10013CV, Corning, Corning, NY, USA). To induce reporter expression, 0.5 µg/mL of doxycycline was added, and cells were incubated at 37 °C with 5% CO_2_ for 24 h. Vertical streaks were generated using a sterile 1000 µL pipette tip. Detached cells were removed by washing three times with Dulbecco’s Phosphate-Buffered saline (DPBS, Fisher, Houston, TX, USA). Cells treated with or without 200 nM Bafilomycin A1 were subsequently observed at 0 and 6 h using a Nikon A1R confocal microscope (Melville, NY, USA).

### 2.9. Statistics Analysis

Statistical analysis was conducted using GraphPad Prism 10 (GraphPad Prism 10.0.2). *p*-values were calculated using Student’s *t*-test or one- or two-way ANOVA, with Tukey correction applied for multiple comparison tests between selected pairs. Data are presented as mean ± standard error of the mean (s.e.m.). *p* < 0.05 was considered statistically significant.

## 3. Results

### 3.1. Validation of a Tandem Fluorescence Reporter System for Focal Adhesion Turnover

**Figure 1:**
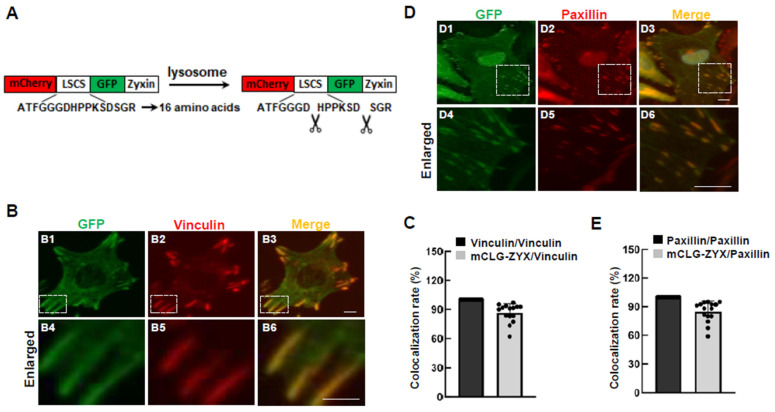
The FA-phagy reporter localizes at focal adhesions. (**A**) The schematic of FA-phagy reporter design. Both mCherry and GFP were fused to the N-terminus of the focal adhesion marker protein zyxin (abbreviated as mCLG-ZYX), with an added linker-specific cleavage site (LSCS). (**B**–**E**) Representative images of HeLa cells expressing the mCLG-ZYX reporter. (**B1**,**D1**) GFP signals from the mCLG-ZYX reporter were enhanced by immunostaining with an anti-GFP antibody (green). (**B2**) Endogenous vinculin and (**D2**) endogenous paxillin were detected by immunostaining using secondary antibodies conjugated with Alexa Fluor 647 (red). (**B3**,**D3**) Merged images of the mCLG-ZYX reporter signals (**B1**,**D1**) and endogenous focal adhesion markers, vinculin (**B2**) or paxillin (**D2**). (**B4**–**B6**,**D4**–**D6**) Higher-magnification views of focal adhesion regions indicated in (**B1**–**B3**) and (**D1**–**D3**). (**C**,**E**) Quantification of co-localization ratios for the mCLG-ZYX reporter co-localized with vinculin (**D**) or paxillin (**E**). *n* = 15 cell counts, 20× objective. Scale bar: 10 µm.

To monitor autophagy-mediated focal adhesion turnover, we utilized a tandem fluorescence reporter system. As shown in Figure 1A, both mCherry and GFP were sequentially fused to the N-terminal of zyxin, a focal adhesion marker protein [23]. Therefore, our reporter is expected to show both red and green fluorescence (merged as yellow signals) at focal adhesions. To detect mCherry release after entry into the lysosome, we added a linker-specific cleavage site (LSCS), as previously described for studying aggrephagy [20]. LSCS is specifically recognized by legumain, a protease residing in lysosomes [21].

The expression of the reporter was confirmed by Western blot against mCherry (Supplemental Figure S1). To examine whether the tandem fluorescence tag interferes with the focal adhesion localization of zyxin, we co-immunostained the reporter with endogenous focal adhesion markers, including paxillin and vinculin [24]. To avoid overlapping signals with mCherry and GFP, endogenous paxillin and vinculin were highlighted using a far-red dye (Alexa 647). As shown in Figure 1B–E, both paxillin and vinculin highly co-localized with the reporter, indicating that the reporter faithfully integrates into endogenous focal adhesion complexes.

### 3.2. The Reporter Responds to Autophagy Stimuli

**Figure 2:**
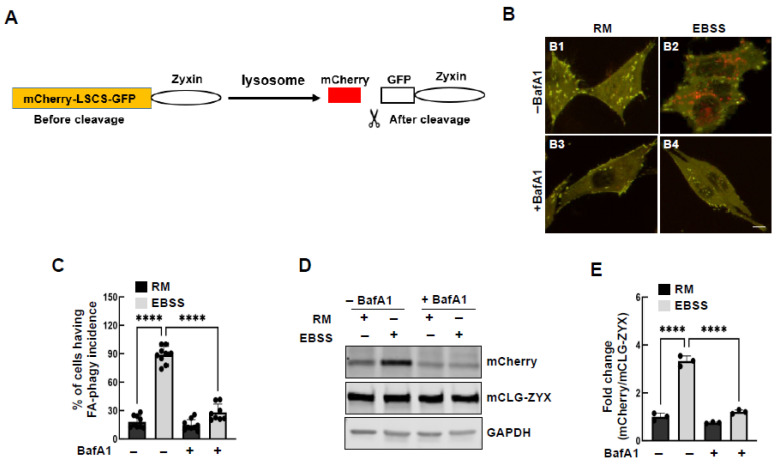
The FA-phagy reporter is specific and sensitive to autophagy stimuli. (**A**) Schematic model of how FA-phagy reporter is processed. The GFP signal is quenched in lysosomes. mCherry-LSCS-GFP-Zyxin is cleaved by lysosomal enzymes to yield the mCherry fragment. LSCS: linker-specific cleavage site. (**B**) Representative live fluorescence images of HeLa cells expressing mCLG-ZYX treated with EBSS for 16 h in the presence and absence of BafA1 (100 nM). (**B1**) Under basal conditions (Rich medium). (**B2**) EBSS treatment for 16 h. (**B3**) Rich medium for 16 h in the presence of bafilomycin A1 (BafA1, 100 nM). (**B4**) EBSS medium for 16 h in the presence of bafilomycin A1 (BafA1, 100 nM) (**C**) The ratio of FA-phagy incidence was quantified from at least 9 different areas (20× objective) and one-way ANOVA followed by Tukey post hoc analysis was used to reveal the statistical difference. (**D**) Western blot analysis of released mCherry from the reporter in the same conditions as shown in (**B**,**C**). Further quantification of the protein level of released mCherry normalized to mCLG-ZYX is shown in (**E**). GAPDH serves as loading control and *n* = 3 independent experiments. **** *p* < 0.0001. Scale bar: 10 µm.

The reporter displays both red and green fluorescence in autophagosomes. However, upon lysosomal entry, the acidic environment quenches the green fluorescence [15], while mCherry remains resistant (Figure 2A). Concurrently, legumain in the lysosome cleaves the LSCS, releasing mCherry from the reporter (Figure 2A). To evaluate the sensitivity of the reporter to autophagy stimuli, we treated cells with EBSS, a condition that is widely used to induce autophagy [25]. EBSS treatment markedly increased the accumulation of red puncta (Figure 2(B1,B2); second vs. first column in Figure 2C), an effect blocked by the lysosomal inhibitor BafA1 (Figure 2(B2,B4); fourth vs. second column in Figure 2C). Consistently, EBSS treatment induced the accumulation of released mCherry fragments, which was abrogated by BafA1 (Figure 2D,E). Since mCherry was released from a portion of the reporter, longer exposure is needed for its detection (Supplemental Figure S2). To evaluate the effect of starvation on overall reporter stability, we monitored GFP-only signals, which are quenched upon delivery to lysosomes. As shown in Supplemental Figure S3, starvation significantly reduced the number of GFP-positive puncta, whereas treatment with bafilomycin A1 prevented this reduction (Supplemental Figure S3). This observation is consistent with prior reports on GFP-tagged reporters in selective autophagy [26]. These findings demonstrate that the reporter is both sensitive and specific to autophagy stimuli.

### 3.3. The Reporter Enters the Autophagy System

**Figure 3:**
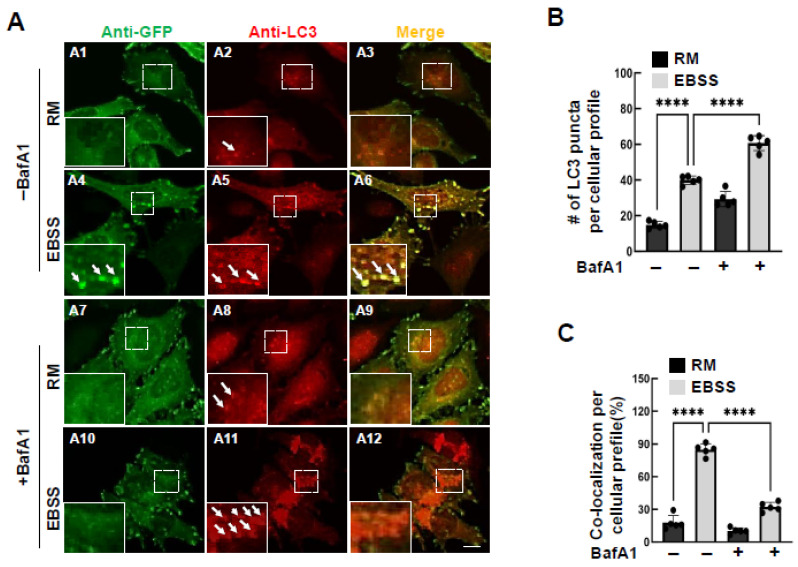
The FA-phagy reporter enters autophagosomes upon autophagy stimulation. (**A**) Representative images of HeLa cells immunostained for endogenous LC3 and the stably expressed mCLG-ZYX reporter. Cells were treated with EBSS for 16 h in the presence or absence of bafilomycin A1 (BafA1, 100 nM). Co-localization between the mCLG-ZYX reporter and LC3 is shown in the merged images (yellow). Each image in this panel is labeled with an identifier (e.g., A1) in the upper-left corner. (**A1**–**A3**) Basal conditions (rich medium). (**A1**) GFP signals from the mCLG-ZYX reporter were enhanced by immunostaining with an anti-GFP antibody (green). (**A2**) Endogenous autophagosome protein LC3 was detected by immunostaining using secondary antibodies conjugated with Alexa Fluor 647 (red). (**A3**) Merged image of the mCLG-ZYX reporter signal (**A1**) and endogenous LC3 (**A2**). (**A4**–**A6**) EBSS treatment for 16 h. (**A4**) GFP signals from the mCLG-ZYX reporter were enhanced by immunostaining with an anti-GFP antibody (green). (**A5**) Endogenous autophagosome protein LC3 was detected by immunostaining using secondary antibodies conjugated with Alexa Fluor 647 (red). (**A6**) Merged image of the mCLG-ZYX reporter signal (**A4**) and endogenous LC3 (**A5**). (**A7**–**A9**) Rich medium for 16 h in the presence of bafilomycin A1 (BafA1, 100 nM). (**A7**) GFP signals from the mCLG-ZYX reporter were enhanced by immunostaining with an anti-GFP antibody (green). (**A8**) Endogenous autophagosome protein LC3 was detected by immunostaining using secondary antibodies conjugated with Alexa Fluor 647 (red). (**A9**) Merged image of the mCLG-ZYX reporter signal (**A7**) and endogenous LC3 (**A8**). (**A10**–**A12**) EBSS treatment for 16 h in the presence of bafilomycin A1 (BafA1, 100 nM). (**A10**) GFP signals from the mCLG-ZYX reporter were enhanced by immunostaining with an anti-GFP antibody (green). (**A11**) Endogenous autophagosome protein LC3 was detected by immunostaining using secondary antibodies conjugated with Alexa Fluor 647 (red). (**A12**) Merged image of the mCLG-ZYX reporter signal (**A10**) and endogenous LC3 (**A11**). (**B**) The number of LC3-positive puncta per cellular profile was calculated from at least five different areas (20× objective) and further quantified by one-way ANOVA followed by Tukey’s post hoc analyses. (**C**) The rate of co-colocalization between mCLG-ZYX and LC3 as indicated in (**A**) was calculated from at least 5 areas (20× objective) and further quantified by one-way ANOVA followed by Tukey’s post hoc analyses. **** *p* < 0.0001. Scale bar: 10 µm.

To examine whether the reporter enters the autophagy pathway, we examined its co-localization with autophagosomes. At basal conditions, we noticed that few autophagosomes were detected (Figure 3(A2)), consistent with previous reports that autophagy remains low in rich medium. However, when cells were starved in EBSS, autophagosome formation increased, and these autophagosomes partially co-localized with the reporter (Figure 3(A4–A6)). BafA1 treatment increased the number of autophagosome as expected (Figure 3(A8,A11),B) and reduced the subcellular localization of the reporter at autophagosomes (Figure 3C). This aligns with the accumulation of focal adhesion reporters in BafA1-treated cells.

**Figure 4:**
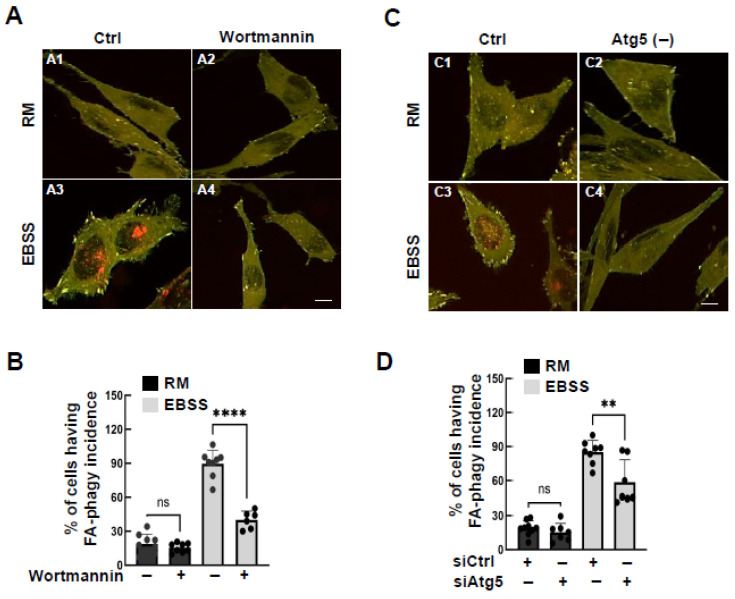
Autophagy biogenesis is required for FA-phagy. (**A**,**B**) Representative live-cell images of HeLa cells expressing mCLG-ZYX reporter treated with EBSS (16 h) vs. RM (rich medium) in the absence and presence of wortmannin (20 nM). (**A1**) Basal conditions (Rich medium). (**A2**) Rich medium for 16 h in the presence of wortmannin (wortmannin, 20 nM). (**A3**) EBSS treatment for 16 h. (**A4**) EBSS medium for 16 h in the presence of wortmannin A1 (wortmannin, 20 nM). The percentage of cells having FA-phagy incidence was calculated from at least 9 different areas (20× objective) and quantified in (**B**). (**C**,**D**) Live imaging of FA-phagy activity based on mCLG-ZYX reporter in HeLa cells treated with siRNAs against *atg5* and the results are quantified in (**D**). (**C1**) Basal conditions (rich medium). (**C2**) Rich medium for 16 h following *atg5* knockdown for 72 h. (**C3**) EBSS treatment for 16 h. (**C4**) EBSS treatment for 16 h following *atg5* knockdown for 72 h. All quantifications represent the percentage of cells having FA-phagy incidence and were analyzed by one-way ANOVA followed by Tukey’s post hoc tests. ** *p* < 0.01, **** *p* < 0.0001. ns: not significant. Scale bar: 10 µm.

To investigate whether autophagosome biogenesis contributes to the observed phenotype, we inhibited autophagosome formation using wortmannin, a class III PI3K inhibitor essential for autophagosome biogenesis [27]. Wortmannin significantly reduced EBSS-induced red puncta accumulation (Figure 4A,B). Additionally, knockdown of essential autophagy genes (*ATG5*, *ATG7*, and *Beclin 1*) similarly suppressed EBSS-induced red puncta formation (Figure 4C; Supplemental Figures S4 and S5). These findings suggest that the processing of the reporter relies on macroautophagy, requiring both autophagosome biogenesis and lysosomal degradation.

**Figure 5:**
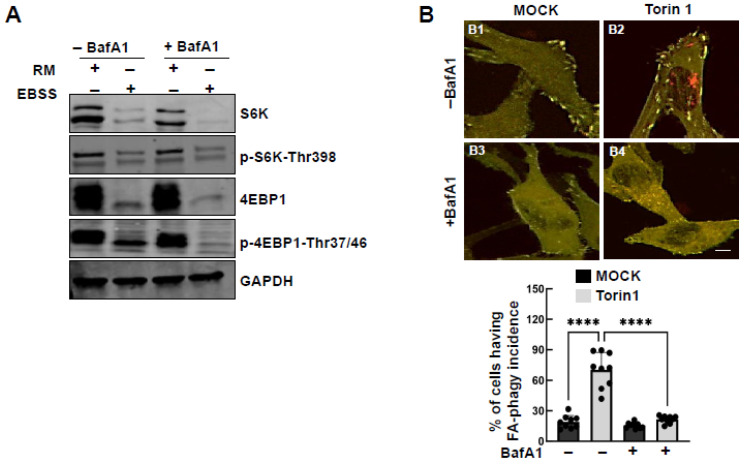
mTOR pathway contributes to starvation-induced FA-phagy. (**A**) Western blot analysis of S6K, p-S6K-Thr398, 4EBP1, and p-4EBP-Thr37/46 in whole cell lysates of HeLa cells expressing the mCLG-ZYX reporter treated with EBSS (16 h) vs. RM (rich medium) in the absence and presence of BafA1 (100 nm). GAPDH serves as the loading control. (**B**) Representative images of HeLa cells expressing mCLG-ZYX reporter treated with Torin 1 (1 μM) vs. MOCK (DMSO) for 16 h. (**B1**) Mock treatment (rich medium with DMSO, 1:1000) for 16 h. (**B2**) Torin1 treatment (1 µM, dissolved in DMSO) in rich medium for 16 h. (**B3**) Mock treatment (rich medium with DMSO, 1:1000) for 16 h in the presence of bafilomycin A1 (BafA1, 100 nM). (**B4**) Torin1 treatment (1 µM, dissolved in DMSO) in rich medium for 16 h in the presence of bafilomy-cin A1 (BafA1, 100 nM). FA-phagy incidence per cellular profile was calculated from at least 9 different areas and quantified. **** *p* < 0.0001. Scale bar: 10 µm.

It is well documented that mTOR signaling is a key regulator of autophagy [28]. Under EBSS treatment, the expression and phosphorylation levels of mTOR substrates S6K and 4EBP1 were downregulated (Figure 5A), consistent with a previous report that mTOR senses the nutrient status in cells. Treatment with Torin 1, an mTOR kinase inhibitor, significantly increased red puncta formation (Figure 5B). These data suggest that mTOR pathway suppression facilitates autophagy-mediated processing of the reporter.

### 3.4. Functional Assessment of the Reporter in Different Cell Types

**Figure 6:**
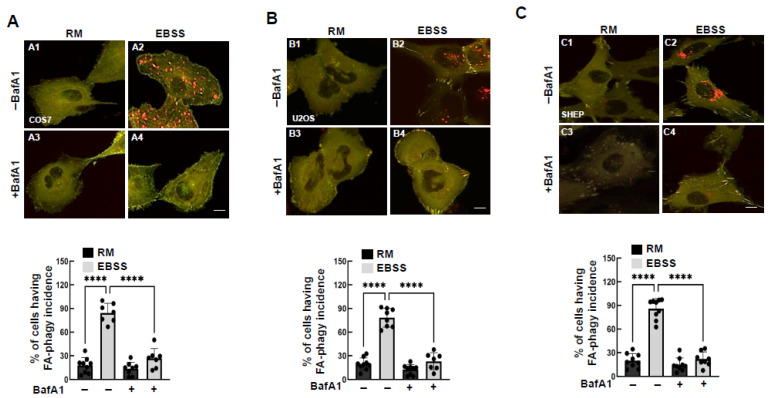
The FA-phagy reporter is applicable in different cells. (**A**–**C**) The mCLG-ZYX reporter expressed in different cell lines such as COS7 in (**A**), U2OS in (**B**), and SHEP in (**C**). (**A1**,**B1**,**C1**) Under basal conditions (Rich medium). (**A2**,**B2**,**C2**) EBSS treatment for 16 h. (**A3**,**B3**,**C3**) Rich medium for 16 h in the presence of bafilomycin A1 (BafA1, 100 nM). (**A4**,**B4**,**C4**) EBSS medium for 16 h in the presence of bafilomy-cin A1 (BafA1, 100 nM). The representative images were taken from different cells. FA-phagy activity was induced in all above cells treated with EBSS (16 h) in the absence and presence of BafA1 (100 nM). FA-phagy incidence per cellular profile was calculated from at least 9 different areas (20× objective) and quantified as shown at the bottom. **** *p* < 0.0001. Scale bar: 10 µm.

To assess the versatility of the reporter, we evaluated its function in various cell lines, including COS7 (monkey fibroblast), U2OS (human osteosarcoma), SHEP (human neuroblastoma), and NIH3T3 (mouse embryonic fibroblast). In all cell lines tested, EBSS treatment induced significant red puncta accumulation, which was blocked by BafA1 (Figure 6A–C; Supplemental Figure S6A). Similarly, mCherry release from the reporter was observed following EBSS treatment but inhibited by BafA1 (Supplemental Figures S6C,D and S7A–C). These results indicate that the reporter is not cell-type-specific and can effectively monitor FA-phagy across diverse cell types.

To demonstrate the application of our reporter in a cellular function, we evaluated its role during cell migration using a wound healing assay, as focal adhesions undergo rapid turnover in this process. With this assay, we validated the utility of our FA-phagy reporter by showing that FA-phagy activity is markedly increased in the migrating front (compare new Supplemental Figure S8(B2,B4)). Consistent with this, the wound area narrowed significantly after 6 h, whereas inhibition of FA-phagy delayed wound closure (compare new Supplemental Figure S8(A2,A4)). These results provide evidence that our reporter effectively assesses FA-phagy activity in the context of cellular functions and may help distinguish physiological from pathological condition.

## 4. Discussion

### 4.1. A Novel Tool for Monitoring FA-Phagy

This study develops a novel tandem fluorescence reporter for monitoring FA-phagy. To our knowledge, this is the first tool capable of tracking the entire flux of FA-phagy, from autophagosome formation to lysosomal degradation, using a red–green fluorescence system combined with a lysosome-specific cleavage site. The reporter faithfully integrated into focal adhesion complexes without interfering with their localization or function. It demonstrated sensitivity and specificity to autophagy stimuli, effectively distinguishing different stages of autophagy-mediated focal adhesion turnover. Importantly, the functionality of the reporter was validated across multiple cell lines, including human, mouse, and monkey cells, highlighting its robustness and broad applicability.

An interesting observation in our study, consistent with reports on other selective autophagy reporters such as ER-phagy and mitophagy reporters [26,29,30], is that only a small fraction of the reporter appears to undergo lysosomal cleavage. One explanation is that the abundance of lysosomal proteases that cleave the linker site is limiting, so only a subset of reporter molecules that reach the lysosome are processed. Equally possible is the fact that the time required to cleave the linker site inside lysosomes is longer than the time required for reporter delivery, which leads to accumulation of intact reporter within lysosomes as observed. Together, these considerations contribute to the partial cleavage we detect and align with published experience using tandem selective autophagy reporters.

In our experiments using the mCherry-GFP-zyxin reporter, starvation caused a marked reduction in GFP-only puncta, consistent with GFP quenching upon lysosomal delivery, whereas this reduction was blocked by bafilomycin A1 (Supplemental Figure S3). Under the same conditions, we observed the expected starvation-dependent increase in the mCherry/GFP ratio and the accumulation of free mCherry fragments (Figure 2B–E). These complementary readouts arise from the mechanistic properties of the tandem reporter: GFP fluorescence is lost in acidic autolysosomes, allowing GFP-only puncta to reflect overall reporter turnover, while mCherry remains stable and thus distinguishes autophagosomal from autolysosomal states. Because mCherry alone cannot report global stability, the appearance of cleaved free mCherry provides a reliable cargo-based endpoint measure of autophagic flux, consistent with established assays [20,21]. Together, these measurements from our reporter system provide internally consistent and mutually reinforcing evidence supporting our conclusions about FA-phagy dynamics.

### 4.2. The Potential Applications of the FA-Phagy Reporter in Health and Disease

The development of this novel FA-phagy reporter has significant implications for advancing our understanding of FA dynamics and their role in cellular processes. By enabling the dynamic observation of FA-phagy, this tool opens avenues for identifying new regulatory mechanisms and signaling pathways involved in focal adhesion turnover. Such insights can lead to the discovery of novel therapeutic targets and further understanding of the molecular events governing FA dynamics.

Given the critical role of FAs in cancer metastasis, this reporter provides cancer researchers with a powerful method to study how FA turnover contributes to cell migration and invasion. Insights gained from such studies could inform the development of anti-metastatic therapies, offering new avenues for combating cancer progression. Moreover, the real-time monitoring capabilities of this tool allow for detailed studies of how FA-phagy is regulated under varying conditions, making it invaluable in deciphering the complexity of tumor biology. FAs are also essential for maintaining vascular integrity, including the blood–brain barrier [30,31,32]. This tool offers vascular biologists a novel method to explore how FA-phagy regulates vascular health and its potential involvement in diseases such as aneurysms and stroke. By enabling the study of FA-phagy in blood–brain barrier and other vascular systems, researchers can better understand the mechanisms underlying vascular dysfunction and identify strategies to mitigate these effects.

Beyond cancer and vascular biology, the reporter’s applicability extends to fields such as mechanobiology, tissue regeneration, and immune cell migration, where FA dynamics are critically involved. This reporter provides a new avenue to identify and characterize molecular mechanisms that are important for these biological processes.

## 5. Future Directions

While the FA-phagy reporter represents a significant advancement, several limitations and opportunities for future development remain. Our findings indicate that inhibition of autophagosome biogenesis, using both pharmacological inhibitors and siRNA-mediated knockdown of *ATG5*, *ATG7*, and *Beclin 1*, consistently reduced FA-phagy activity, supporting the conclusion that macroautophagy is the primary mechanism driving focal adhesion turnover. Nevertheless, we cannot fully exclude the possibility that a fraction of the reporter may undergo lysosomal degradation through microautophagy, especially given that our current system relies on reporter overexpression and partial inhibition of autophagosome formation. Future studies employing endogenous tagging of focal adhesion proteins and CRISPR-Cas9-mediated knockout of essential autophagy genes will be necessary to distinguish between macroautophagic and microautophagic contributions under physiological conditions.

Although the reporter enables robust monitoring of focal adhesion turnover via lysosomal degradation, the molecular mechanisms underlying selectivity in FA-phagy were not examined in this study. Different cargo receptors have been reported in various cell types [13], suggesting that FA-phagy selectivity could be cell-type-dependent. In future studies, to further leverage this reporter for investigating cargo recognition mechanisms in FA-phagy, we plan to employ complementary approaches, including candidate-based inactivation of reported cargo receptors as well as unbiased functional genomic screens (e.g., CRISPR-Cas9). These strategies can also be extended to other cell types, consistent with broader research goals in areas such as vascular and cancer biology.

The use of EBSS-induced starvation is a harsh method, although it is widely used for inducing autophagy in various cell types. Exploring more physiological stimuli, such as small-molecule activators or inhibitors, or biomechanical changes like altered flow, could provide a more refined understanding of FA-phagy dynamics. These approaches would better mimic in vivo conditions, increasing the translational relevance of the findings.

Although validated in several cell lines, the performance of this reporter in primary cells and specialized cell types needs further investigation to ensure its broad applicability across biological systems. Moreover, future work should focus on developing viral delivery systems, such as adeno-associated virus-based methods, to introduce the reporter into animal models. This would allow real-time monitoring of FA-phagy under physiological and pathological conditions in tissues such as the vascular systems with normal and altered flow environments.

In addition, future studies could extend the utility of our FA-phagy reporter by integrating time-lapse single-cell imaging and flow cytometry approaches. Live imaging would enable real-time tracking of reporter turnover in individual cells. Using the doxycycline-inducible system, doxycycline withdrawal would allow monitoring of the degradation kinetics of specific FA-phagy puncta over time. In parallel, applying a panel of autophagy-modulating agents could reveal how FA-phagy dynamics are regulated by distinct autophagic pathways. Flow cytometry would provide a complementary, population-level quantification by simultaneously measuring green (GFP) and red (mCherry) fluorescence signals in single cells, thereby offering a high-throughput means to assess FA-phagy regulation under multiple experimental conditions. By addressing these limitations and expanding the scope of its applications, the FA-phagy reporter may continue to help elucidate the role of FA dynamics in health and disease.

## 6. Conclusions

In summary, we developed and validated a novel tandem fluorescence reporter that sensitively monitors the complete FA-phagy flux, from autophagosome engagement to lysosomal degradation of focal adhesion complexes. By combining a red–green fluorescence system with a lysosome-specific cleavage site and an endogenous focal adhesion protein, this reporter overcomes limitations of LC3-based assays and provides a dynamic, flux-based readout. Using this tool, we show that FA-phagy is induced by starvation and mTOR inhibition, requires core autophagy machinery, is conserved across cell types, and is spatially enhanced during cell migration. These results support a regulated, biologically relevant role for autophagy in focal adhesion turnover and establish this reporter as a versatile platform for studying its mechanisms and functional consequences.

## Data Availability

The original contributions presented in this study are included in the article/Supplementary Materials. Further inquiries can be directed to the corresponding authors.

## Supplementary Materials

The following supporting information can be downloaded at https://www.mdpi.com/article/10.3390/cells15030306/s1, Figure S1: Validation of mCLG-ZYX reporter expression. Figure S2: Expression levels of mCLG-ZYX reporter at different exposure. Figure S3: Bafilomycin A1 promotes GFP accumulation. Figure S4: The knockdown effects of *atg7* and *beclin 1* on FA-phagy. Figure S5: The knockdown effectiveness of *atg5*, *atg7*, and *beclin 1*. Figure S6: The sensitivity of the FA-phagy reporter in a mouse cell line. Figure S7: mCherry fragment accumulation in different cell lines. Figure S8: FA-phagy reporter is activated in the migrating front.
