# Supplementary material for: Tracking Focal Adhesion Turnover: A Novel Reporter for FA-Phagy Flux
Source: Cells. 2026 Feb 6;15(3):306. doi: 10.3390/cells15030306 (PMC12896405; doi:10.3390/cells15030306)
Supplement: Supplementary file 1 [file cells-15-00306-s001.zip › cells-4055288-supplementary.pdf]

Supplemental Figures:

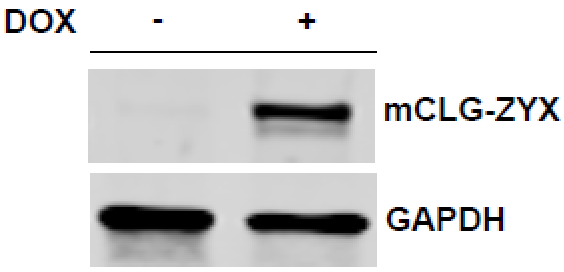

Supplemental Figure S1: Validation of mCLG-ZYX reporter expression

Western blot analysis of whole cell lysates from HeLa cells containing inducible mCLG-ZYX reporter in the absence and presence of doxycycline (500 ng/ml, 48 hours).

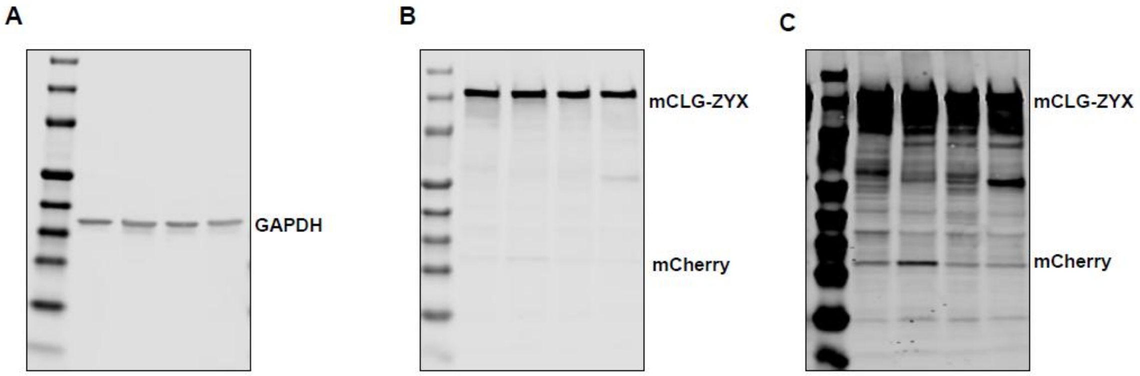

Supplemental Figure S2: Expression levels of mCLG-ZYX reporter at different exposure.

(**A-C**) Western blot analysis of whole cell lysates from HeLa cells expressing mCLG-ZYX reporter by antibodies against GAPDH (A) or mCherry at short exposure time (B) or long exposure time (C).

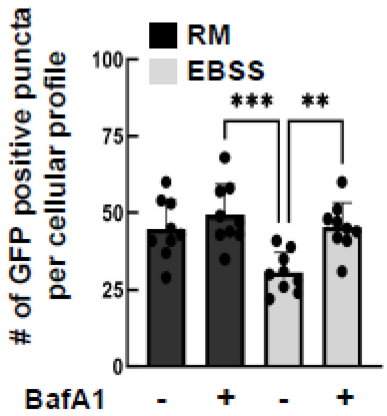

Supplemental Figure S3: Bafilomycin A1 promotes GFP accumulation.

FA-phagy activity was induced in Hela cells treated with EBSS (16 hours) in the absence and presence of BafA1 (100 nM). GFP puncta incidence per cellular profile was calculated from at least 9 different areas (20X objective) and quantified. **p<0.01, ***p<0.001. Scale bar: 10 µm.

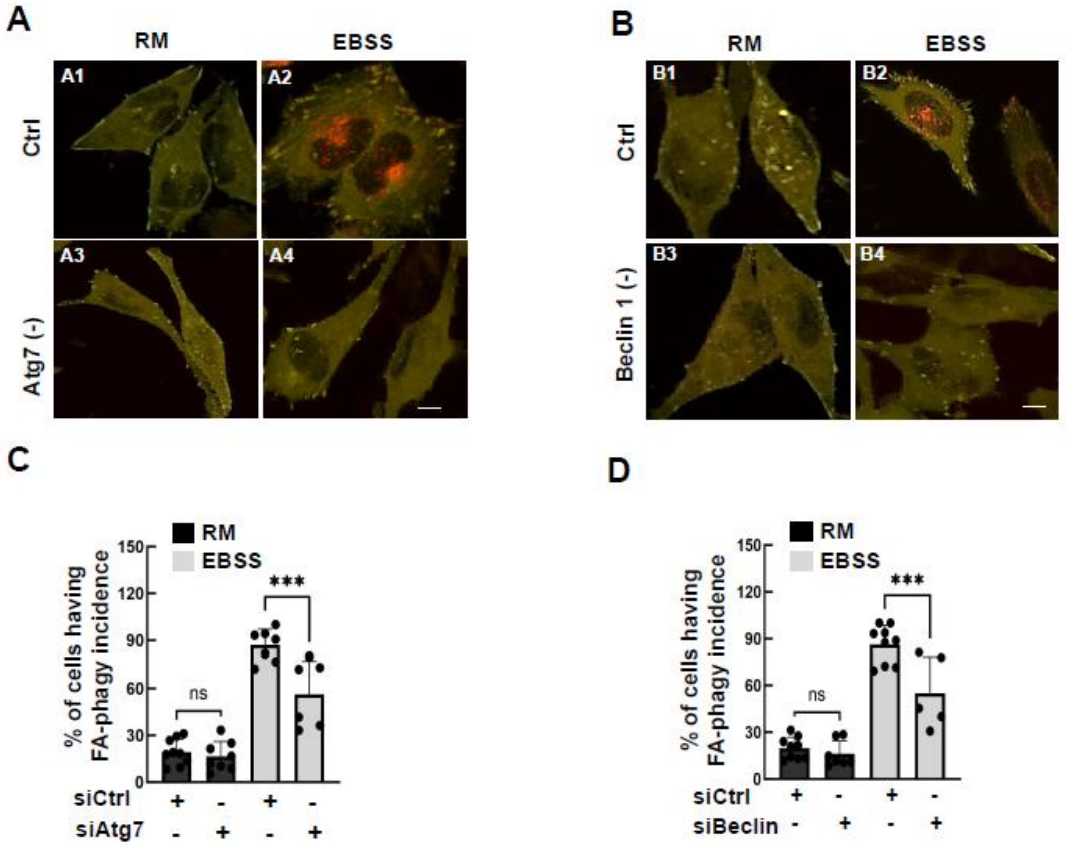

Supplemental Figure S4: The knockdown effects of atg7 and beclin 1 on FA-phagy. (**A-B**) Representative live images were shown of HeLa cells expressing mCLG-ZYX reporter treated with siRNAs against atg7 or beclin 1 (10 nmol for each, 48 hours) vs. control siRNA followed by EBSS treatment (16 hours). (**C-D**) mCherry puncta incidence per cellular profile was calculated from at least 9 different areas (20X objective) and quantified. ***$p<0.001$. Scale bar: 10 μm.

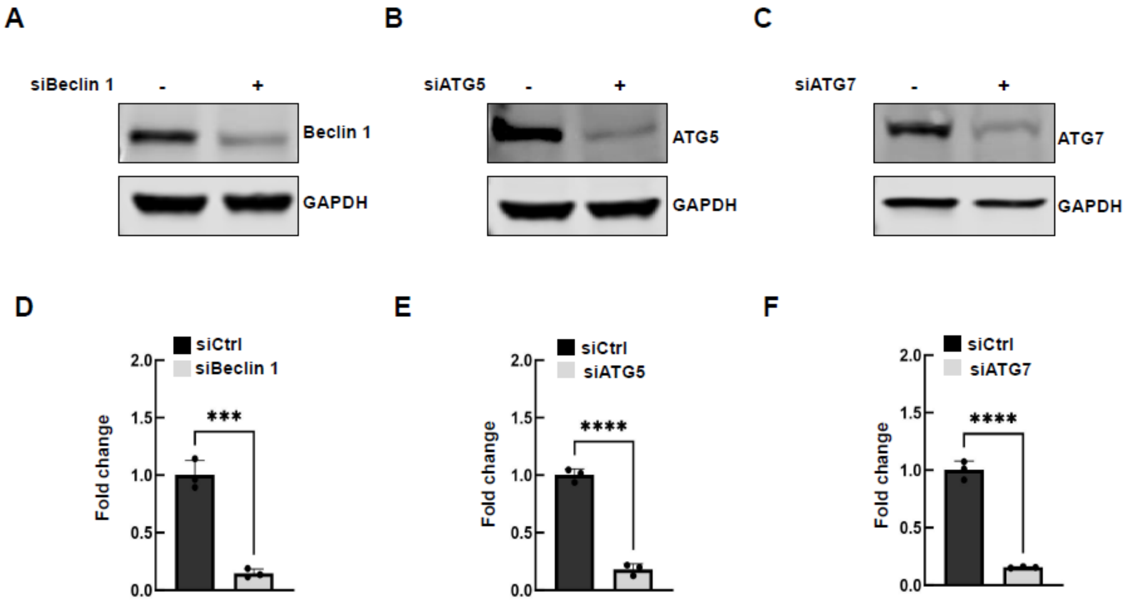

Supplemental Figure S5: The knockdown effectiveness of atg5, atg7, and beclin 1

(A-F) Western blot analysis of whole cell lysates from HeLa cells expressing mCLG-ZYX reporter treated with different siRNAs against beclin 1, atg5, or atg7 (10 nmol for each, 48 hours). The expression level of each marker was normalized to GAPDH, and three independent experiments were performed. Fold change was compared by Student-t test in (D), (E) and (F). ***$p<0.001$, ****$p<0.0001$.

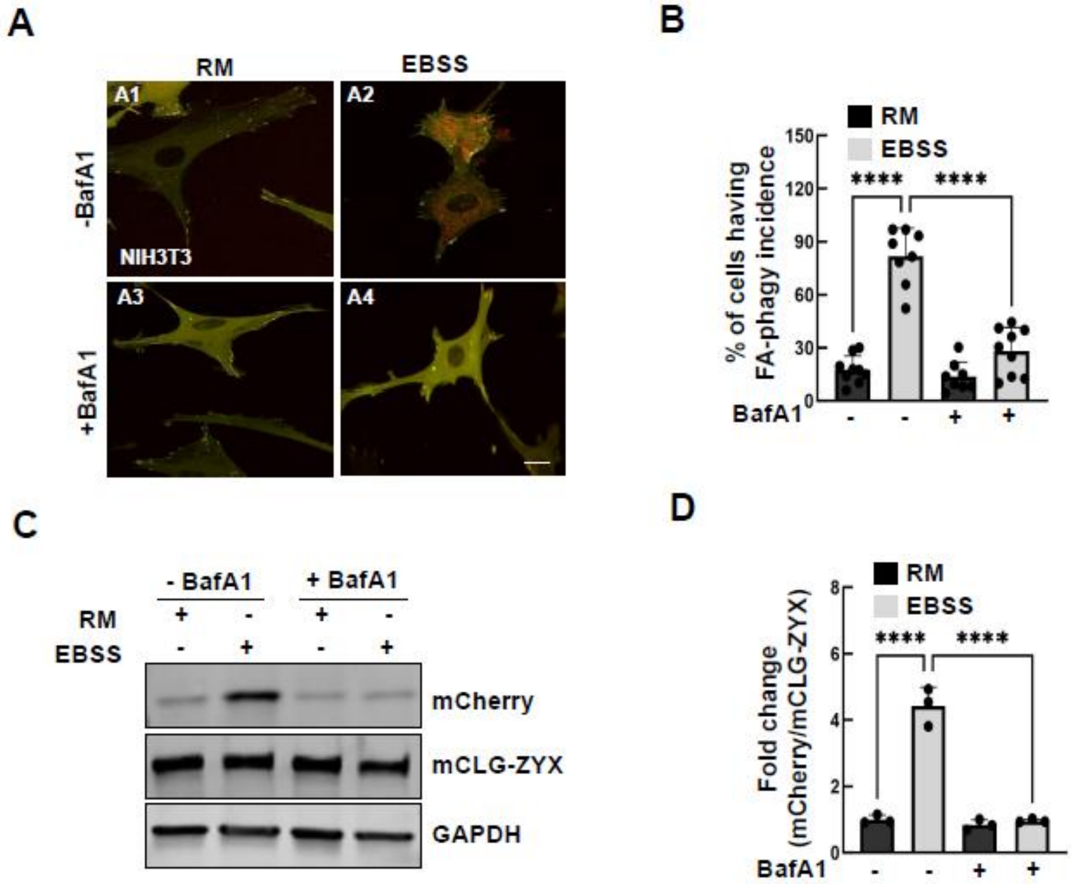

Supplemental Figure S6: The sensitivity of the FA-phagy reporter in a mouse cell line

(**A-B**) FA-phagy activity was induced in NIH3T3 cells treated with EBSS (16 hours) in the absence and presence of BafA1 (100 nM). FA-phagy incidence per cellular profile was calculated from at least 9 different areas (20X objective) and quantified as shown at the bottom. ****$p<0.0001$. Scale bar: 10 µm. (**C-D**) The level of released mCherry was revealed by Western blot analysis of whole cell lysates from NIH3T3 cells treated with EBSS (16 hours) in the absence and presence of BafA1 (100 nm). The ratio of mCherry to mCLG-ZYX was quantified by one-way ANOVA followed by Tukey post hoc analyses

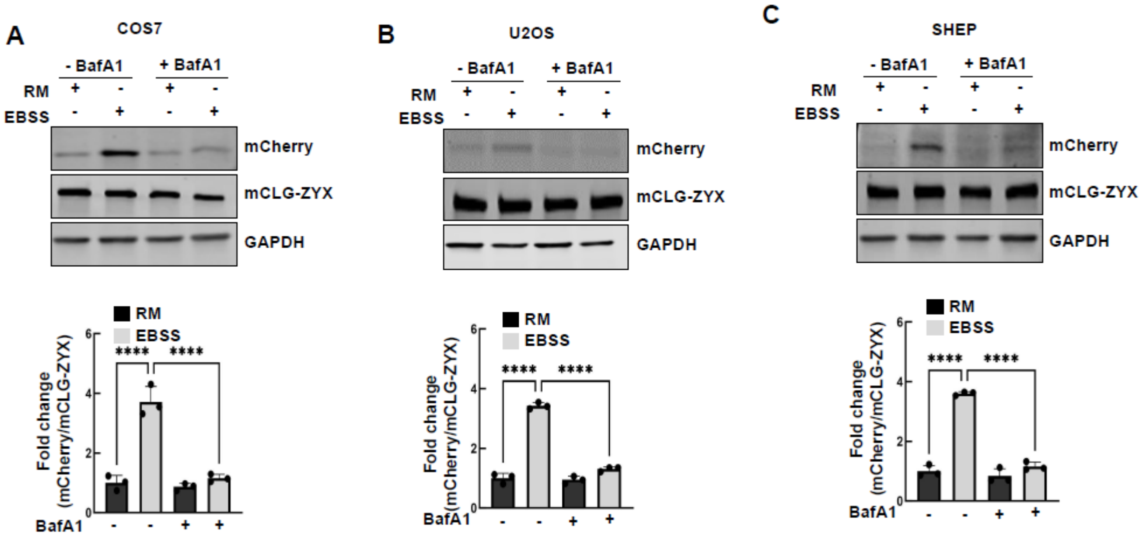

Supplemental Figure S7: mCherry fragment accumulation in different cell lines

(A-C) The level of released mCherry was revealed by Western blot analysis of whole cell lysates from COS7 (A), U2OS (B) and SHEP (C) treated with EBSS (16 hours) in the absence and presence of BafA1 (100 nm). The ratio of mCherry to mCLG-ZYX was quantified by one-way ANOVA followed by Tukey post hoc analyses. ****p<0.0001.

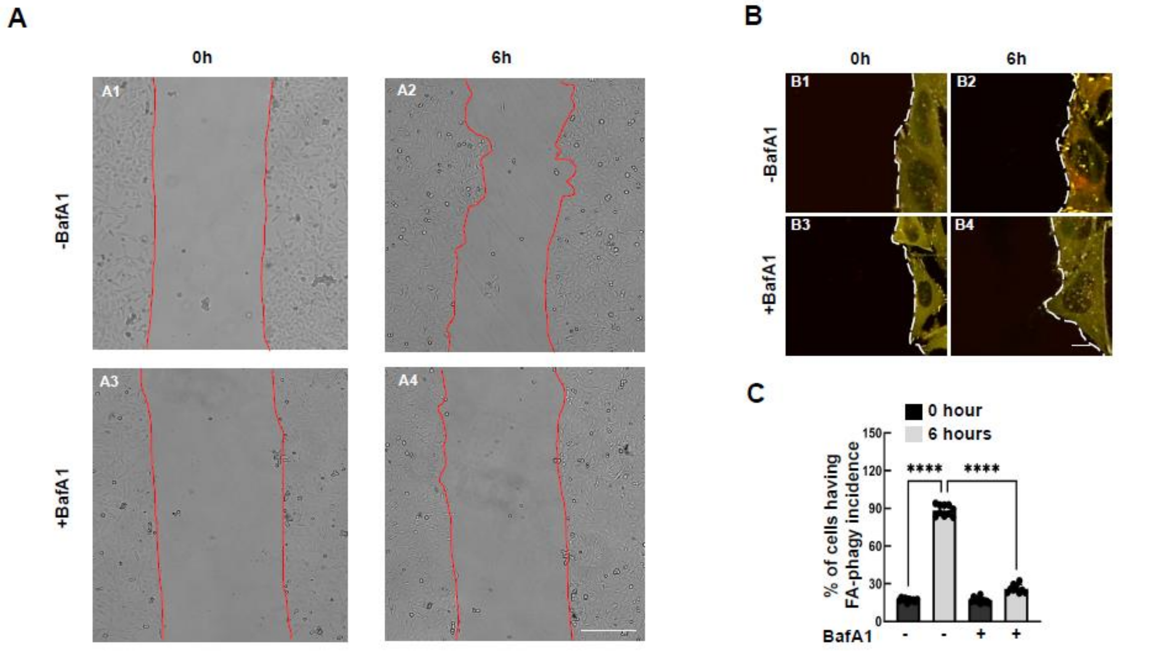

Supplemental Figure S8: FA-phagy reporter is activated in the migrating front

(A) Wounds generated with a 1000 µm tip showed a significant decrease in wound area after 6 hours. In contrast, BafA1 treatment resulted in only a slight reduction in wound area at the

same time point. (B–C) FA-phagy was induced after 6 hours in both the presence and absence of BafA1, coinciding with wound healing. Percentage of cells having FA-phagy incidence was calculated from at least 9 different fields (20× objective) and quantified as shown below. The comparison was analyzed by one-way ANOVA followed by Tukey's post hoc test. ****$p<0.0001$.
